# *Plasmodium knowlesi* Malaria in Children

**DOI:** 10.3201/eid1705.101489

**Published:** 2011-05

**Authors:** Bridget E. Barber, Timothy William, Mohammad Jikal, Jenarun Jilip, Prabakaran Dhararaj, Jayaram Menon, Tsin W. Yeo, Nicholas M. Anstey

**Affiliations:** Author affiliations: Menzies School of Health Research, Darwin, Northern Territory, Australia (B.E. Barber, T.W. Yeo, N.M. Anstey);; Charles Darwin University, Darwin, Northern Territory (B.E. Barber, T.W. Yeo, N.M. Anstey);; Queen Elizabeth Hospital, Kota Kinabalu, Sabah, Malaysia (B.E. Barber, T. William, J. Menon);; Sabah Department of Health, Kota Kinabalu (T. William, M. Jikal, J. Jilip, J. Menon);; Kudat District Hospital, Kudat, Sabah, Malaysia (P. Dhararaj);; Royal Darwin Hospital, Darwin (T.W. Yeo, N.M. Anstey)

**Keywords:** malaria, Plasmodium knowlesi, children, Plasmodium falciparum, parasites, Malaysia, research

## Abstract

*Plasmodium knowlesi* can cause severe malaria in adults; however, descriptions of clinical disease in children are lacking. We reviewed case records of children (age <15 years) with a malaria diagnosis at Kudat District Hospital, serving a largely deforested area of Sabah, Malaysia, during January–November 2009. Sixteen children with PCR-confirmed *P. knowlesi* monoinfection were compared with 14 children with *P. falciparum* monoinfection diagnosed by microscopy or PCR. Four children with knowlesi malaria had a hemoglobin level at admission of <10.0 g/dL (minimum lowest level 6.4 g/dL). Minimum level platelet counts were lower in knowlesi than in falciparum malaria (median 76,500/µL vs. 156,000/μL; p = 0.01). Most (81%) children with *P. knowlesi* malaria received chloroquine and primaquine; median parasite clearance time was 2 days (range 1–5 days). *P. knowlesi* is the most common cause of childhood malaria in Kudat. Although infection is generally uncomplicated, anemia is common and thrombocytopenia universal. Transmission dynamics in this region require additional investigation.

The simian malaria parasite *Plasmodium knowlesi* is increasingly recognized as a frequent cause of potentially fatal human malaria in adults in Malaysian Borneo (*1**–**4*). The infection has also been reported in peninsular Malaysia (*5*) and in other Southeast Asian countries, including Thailand (*6**,**7*), Myanmar (*8**,**9*), Vietnam (*10*), the Philippines (*11*), Indonesian Borneo (*12**–**14*), and Singapore (*15**,**16*). Until recently, *P. knowlesi* had been almost uniformly misdiagnosed by microscopy as *P. malariae* because of its morphologic similarities, leading to underestimations of prevalence (*1**,**17*). Accurate diagnosis therefore requires molecular methods.

The clinical and laboratory features of *P. knowlesi* infections in adults have been described in Kapit, Sarawak, where 107 (70%) of 152 adults with malaria were infected with *P. knowlesi* (*3*). Although *P. knowlesi* malaria was diagnosed in 8 children, the clinical and laboratory features were not described. All previously reported *P. knowlesi* infections that caused clinical disease have been in adults (*1**,**2**,**6**,**8**,**11**–**13**,**15**,**18**–**20*). In malaria caused by *P. falciparum* (*21*) and *P. vivax* (*22*), the 2 species that cause the greatest number of human malaria cases, well-described differences exist between adults and children in terms of the clinical epidemiology, disease spectrum, and laboratory manifestations of disease. We report the demographic, clinical, and laboratory features of *P. knowlesi* infection in children in Kudat, Sabah, a rural coastal farming area with little remaining primary rainforest, an epidemiologic setting that contrasts with the previously described forested areas of Sarawak.

## Methods

### Study Setting

The study was conducted at Kudat District Hospital (KDH) on the northeast tip of Sabah, Malaysia, a coastal rural area which has been largely deforested. KDH services 5 subdistricts (Tigapapan, Dualog, Matunggung, Tambuluran, and the island of Banggi), with a total population of 85,000 persons. The Rungus are the most common ethnic group on the mainland, and minority groups include ethnic Chinese, Bajaus, Dusuns, and Balabaks.

Ministry of Health policy in Sabah requires that all patients with a blood film result that indicates malaria be admitted to the hospital and discharged only after smear results are negative for 2 consecutive days. Since January 2009, in response to increasing reports of *P. knowlesi* infections and the difficulties of diagnosing this species by microscopy, it became policy at KDH to send slides that had been determined by microscopy to show presence of *P. malariae* to the Sabah State Reference Laboratory for PCR confirmation. In addition, KDH sends ≈15% of all blood films positive for other *Plasmodium* spp. for PCR confirmation.

### Retrospective Case Review

We retrospectively searched laboratory microscopy records for all blood smear results positive for *Plasmodium* spp. during January 1–November 30, 2009. The patient’s age, sex, ethnicity, and address were recorded for all positive samples. Microscopy results were matched with PCR results.

Medical records were retrieved for all children <15 years of age who had received a diagnosis of malaria on the basis of microscopy results. Demographic, clinical, and laboratory details were extracted by using standardized data forms, which also included disease response to antimalarial treatment. Parasite clearance time was defined as the number of days until negative smear. Anemia and severe anemia were defined as hemoglobin levels <11 g/dL and <7.1 g/dL (*3*), respectively.

### Laboratory Procedures

Blood films were examined by experienced laboratory microscopists at KDH with the parasite count being classified in most on a scale of 1 to 4 (1 = 4–40 parasites/µL, 2 = 41–400 parasites/µL, 3 = 401–4,000 parasites/µL, 4 = >4,000 parasites/µL), with accurate quantitation per microliter being recorded for most blood films that showed *P. knowlesi,* but for only a limited number that showed *P. falciparum*. Hemoglobin level and leukocyte and thrombocyte counts were measured on site by using automated systems (Sysmex XT1800 [Sysmex Corp., Mundelein, IL, USA] and CELL-DYN Sapphire [Abbott Diagnostics, Abbott Park, IL, USA]) At the Sabah State Reference Laboratory, parasite DNA was extracted, and nested PCR was performed for *P. falciparum, P. vivax, P. malariae, P. ovale*, and *P. knowlesi* by methods described (*1**,**23*).

### Statistical Analysis

Data were analyzed by using Stata statistical software, version 10 (StataCorp LP, College Station, TX, USA). Proportions were compared by using the χ^2^ or Fisher exact test. Normally distributed and non–normally distributed variables were compared by using Student *t* test and Wilcoxon rank-sum test, respectively. For comparison between *P. falciparum* and *P. knowlesi* cases*,* the analysis included children with PCR-confirmed *P. knowlesi* infection, and children with *P. falciparum* infection diagnosed by either microscopy or PCR. Children with mixed *Plasmodium* infections were excluded from analysis, as were children whose medical records could not be located.

## Results

### Malaria in All Age Groups

**Figure 1:**
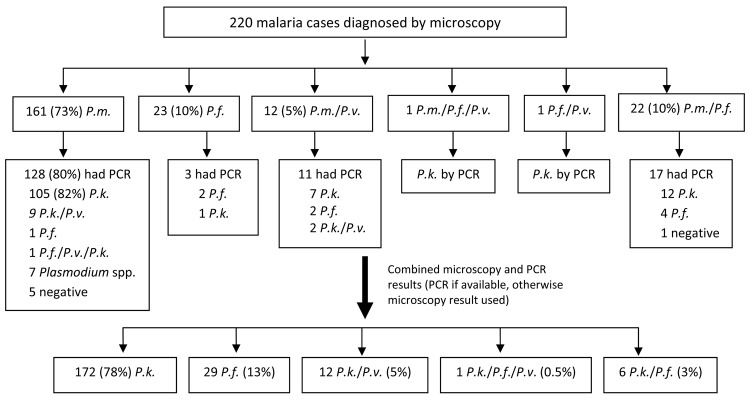
Distribution of malaria cases diagnosed by microscopy and PCR among all age groups, Kudat, Malaysia, January 1–November 30, 2009. *P.m.*, *Plasmodium malariae*; *P.f., P. falciparum*; *P.v., P. vivax*; *P.k., P. knowlesi*; neg, negative.

From January 1 through November 30, 2009, 220 patients at KDH were given a diagnosis of malaria on the basis of microscopy results (Figure 1). Of these, 196 (89%) had *P. malariae* monoinfection or mixed infection. PCR was performed on samples from 157 (80%) of these patients and results were positive for *P. knowlesi* in 137 (87%); 125 (91%) of these were *P. knowlesi* monoinfections. For the remaining 20 patients who had been given a diagnosis of *P. malariae* monoinfection or mixed infection by microscopy, PCR found undifferentiated *Plasmodium* spp., negative results for *Plasmodium* spp., and 7 cases of *P. falciparum* infection.

To estimate the final numbers of malaria species, we used positive PCR results when possible and microscopy results when PCR had not been performed (replacing *P. malariae* with *P. knowlesi*). Microscopy results were also used if PCR result was negative (5 *P. knowlesi* infections and 1 *P. knowlesi/P. falciparum* infection) or if PCR result was positive for *Plasmodium* spp. (7 *P. knowlesi* infections). Using this method, we found 172 (78%) *P. knowlesi* monoinfections, 29 (13%) *P. falciparum* monoinfections, and 19 (9%) mixed infections (Figure 1). A greater proportion of patients with *P. knowlesi* malaria were male (123/172 [72%]) than were those with *P. falciparum* malaria (15/29 [52%]; p = 0.03). Median age was higher for those with *P. knowlesi* infection (median 32 years, interquartile range [IQR] 19–49 years) than for those with *P. falciparum* infection (median 11 years, IQR 6–30 years).

### Malaria in Children

**Figure 2:**
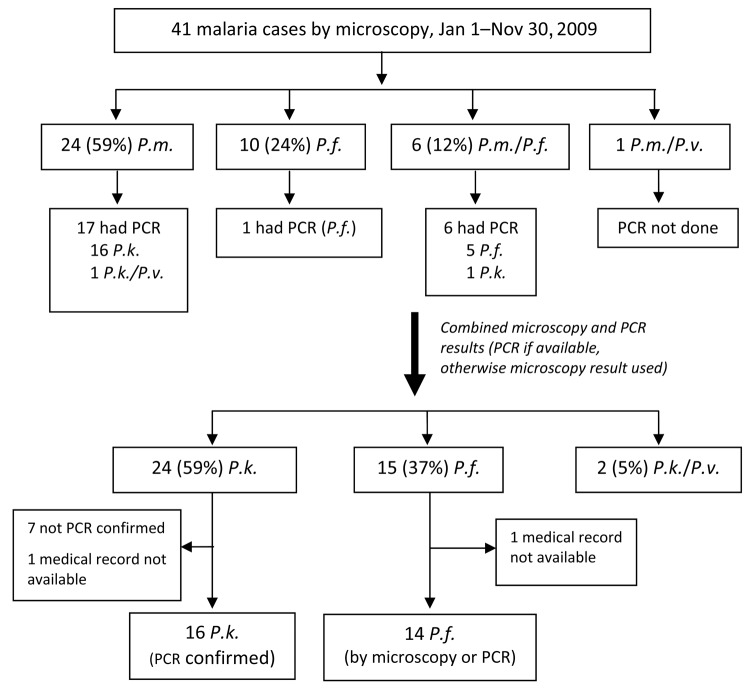
Distribution of malaria cases diagnosed by microscopy and PCR among children <15 years of age, Kudat, Malaysia, January 1–November 30, 2009. *P.m.*, *Plasmodium malariae*; *P.f., P. falciparum*; *P.v., P. vivax*; *P.k.,* *P. knowlesi*.

Of 220 patients with positive results by microscopy, 41 (19%) were <15 years of age. Microscopy showed that 24 (59%) children had *P. malariae* monoinfection, 10 (24%) had *P. falciparum* monoinfection, and 7 (17%) had mixed infections (Figure 2). Samples from 17 children with *P. malariae* monoinfection underwent PCR; 16 (94%) showed *P. knowlesi,* and 1 showed mixed *P. knowlesi/P. vivax* infection. Again, final numbers of malaria species were estimated by using PCR results (if available) or microscopy (if PCR had not been performed). Accordingly, 24 cases (59%) were *P. knowlesi*, 15 (37%) were *P. falciparum,* and 2 were mixed *P. knowlesi/P. vivax* infections. Children thus represented 14% (24/172) of all cases of *P. knowlesi* monoinfection.

Further analysis was confined to those children with PCR-confirmed *P. knowlesi* monoinfection and children with *P. falciparum* monoinfection diagnosed by either microscopy or PCR. Medical records were unavailable for 2 children (1 with *P. knowlesi* and 1 with *P. falciparum* infection), leaving 16 children with PCR-confirmed *P. knowlesi* infection and 14 with *P. falciparum* infection for comparison (Table 1).

**Table 1:** Demographic data, clinical features of infection, and laboratory values in 14 cases of *Plasmodium falciparum* malaria and 16 cases of PCR-confirmed *P. knowlesi* malaria in children, Kudat, Malaysia, January 1–November 30, 2009*

Characteristic	*P. knowlesi*, n =16	*P. falciparum*, n =14	p value
Age, y			
Mean (SD)	8.9 (2.5)	5.4 (3.2)	**0.002**
Range	4–14	0.75–11	
Male sex, no. (%)	8 (50)	7 (50)	1.00
Living in Banggi, no. (%)	0 (0)	10 (71.4)	**<0.001**
Duration of fever, d, median (IQR)	4 (3–6)	6.5 (4–7)	0.12
Examination findings at admission			
Temperature, ^o^C, median (IQR)	37.9 (37.0–39.1)	37.0 (37.0–37.4)	0.07
Heart rate, beats/min, mean (SD)	110 (24)	119 (15)	0.24
Respiratory rate, beats/min, mean (SD)	27.8 (3.9)	32 (3.8)	**0.01**
Systolic blood pressure, mm Hg, mean (SD)	105 (8.3)	98 (11.2)	0.06
Laboratory results			
Hemoglobin d 0, g/dL, median (IQR)	10.7 (10.0–11.4)	8.5 (6.1–10.5)	**0.001**
Hemoglobin minimum level, g/dL, median (IQR)	9.7 (8.4–10.2)	7.15 (6.1–9.3)	**0.035**
Day of hemoglobin minimum level, mean (SD)	2.6 (1.03)	1.5 (1.3)	**0.022**
Lymphocyte count d 0, × 10^3^/µL, median (IQR)	2.0 (1.7–2.3)	2.5 (2.0–4.6)	0.09
Lymphocyte minimum level, × 10^3^/µL, median (IQR)	1.6 (1.3–2.2)	2.4 (1.9–3.9)	**0.01**
Day of lymphocyte minimum level, median (IQR)	1 (0–1)	1.5 (0–3)	0.19
Thrombocyte count d 0, × 10^3^/µL, median (IQR)	89.5 (72.0–118.5)	171.5 (98–271)	**0.038**
Thrombocyte count minimum level, × 10^3^/µL, median (IQR)	76.5 (68.5–110.0)	156 (98–227)	**0.014**
Day of thrombocyte count minimum level, median (IQR)	1 (1–1)	0.5 (0–2)	0.67

*IQR, interquartile range. **Boldface** indicates statistical significance.

### Demographic Characteristics of Children with *P. knowlesi* Malaria

Half of the children with PCR-confirmed *P. knowlesi* malaria were male (Table 1). The mean age was 8.9 (95% confidence interval 7.6–10.3) years. The youngest child with *P. knowlesi* infection was 4 years; all others (15/16; 94%) were 7–14 years of age. In contrast, children with *P. falciparum* malaria were significantly younger, with a mean age of 5.4 years (95% confidence interval 3.5–7.3; range 9 months–11 years; p = 0.002).

### Clinical Features of *P. knowlesi* and *P. falciparum* Malaria

All children had fever or a history of fever. The duration of fever before hospital admission appeared shorter with knowlesi malaria than with falciparum malaria (median 4 days vs. 6.5 days), although this difference was not statistically significant. Although documentation of clinical features was limited, no child was in a state of shock, and no child was reported to be dyspneic, to have bleeding complications, or to have any other clinical feature or laboratory results that indicated severe malaria (*24*). None of the children who had either knowlesi or falciparum malaria died.

#### Anemia

In 11 (69%) children with *P. knowlesi* malaria, the parasite density was accurately determined at hospital admission, with a median of 2,240 (IQR 480–7,200) parasites/µL. Only 2 children with *P. falciparum* malaria had parasite densities accurately determined at admission (200 and 1,600 parasites/µL). Anemia was common with *P. knowlesi* and *P. falciparum* infections. At admission, 9 (56%) children with *P. knowlesi* infection were anemic (hemoglobin level <11.0 g/dL), and 4 (25%) had a hemoglobin level <10 g/dL. All children with *P. knowlesi* infection had a hemoglobin minimum level <11.0 g/dL, and 10 (63%) had a hemoglobin minimum level <10 g/dL. The lowest hemoglobin level was found in an 8-year-old boy: 8.3 g/dL at admission, 6.4 g/dL on day 3, and 7.2 g/dL on day 6, the day before discharge. His parasite count at admission (14,400 parasites/µL) was the highest recorded in this study.

In addition, severe anemia (hemoglobin 6.0 g/dL at admission; minimum level 4.9 g/dL on day 1 requiring transfusion) occurred in a second child with positive results for *P. knowlesi* by microscopy (this child was not included in the final analysis because of lack of PCR confirmation). Among children with *P. knowlesi* infection, a lower hemoglobin minimum level was associated with higher parasite density at admission (p = 0.001). All children with *P. falciparum* infection had hemoglobin levels at admission <11.0 g/dL; 10 (71%) had hemoglobin levels <10 g/dL, and 5 (36%) had severe anemia (hemoglobin <7.1 g/dL). Severe anemia developed in a sixth child after admission. Four children received transfusions, with hemoglobin levels of 4.8–6.1 g/dL at admission. Children with *P. knowlesi* malaria took longer to reach their hemoglobin minimum level than did children with *P. falciparum* malaria (2.6 vs 1.5 days; p = 0.02).

#### Thrombocytopenia

All children with *P. knowlesi* malaria had thrombocytopenia. Fifteen children (94%) had a platelet count <150,000/µL at admission, and the remaining child exhibited thrombocytopenia within 1 day. The lowest platelet count recorded was 28,000/µL. Platelet count was not correlated with hemoglobin level. Thrombocytopenia was less common in children with *P. falciparum*; 7 (50%) had platelet counts <150,000/µL at admission or during hospitalization and the lowest platelet count was 47,000/µL. Children with *P. knowlesi* malaria had a lower lymphocyte count minimum than those with *P. falciparum* malaria (1.6 vs. 2.4 × 10^3^/µL; p = 0.01).

### Response to Treatment

Most (81%) children with *P. knowlesi* malaria were given oral chloroquine and primaquine for 3 days, and these children had a median parasite clearance time of 2 days (Table 2). The longest parasite clearance time with chloroquine and primaquine was 5 days in the aforementioned 8-year-old boy with the highest admission parasitemia level (14,400/µL) and the lowest minimum hemoglobin level (6.4 g/dL). Three children with *P. knowlesi* infection were given oral quinine, and parasites cleared within 2 days. Among the 11 children with *P. knowlesi* and parasite densities accurately assessed at admission, the correlation between parasite density and parasite clearance time was significant (p = 0.002).

**Table 2:** Malaria treatment according to *Plasmodium* species and response to treatment in children, Kudat, Malaysia, January 1–November 30, 2009*

Species and treatment regimen	No. (%) patients	Days until negative smear†
*P. knowlesi,* n = 16		
3 days CQ/PQ	13 (81)	2 (2–3, 1–5)
Oral Q	2 (13)	2, 2
IV Q	1 (6)	2
All		2 (2–3, 1–5)
*P. falciparum,* n = 14		
3 days CQ/PQ → oral Q	2 (14)	5, 5
Oral Q → AL	1 (7)	3
SP/PQ → AM	1 (7)	8
SP/PQ	2 (14)	1, 2
SP/PQ → CQ/Q → AL	1 (7)	3
SP/PQ → CQ/IV Q → AL	1 (7)	5
SP/PQ → CQ/IV Q → oral Q	1 (7)	3
SP/PQ → oral Q → IV Q → oral Q	1 (7)	16
IV Q → oral Q	2 (14)	1, 10
IV Q → oral Q/PQ	1 (7)	11
Oral Q/PQ → AM	1 (7)	10
All		5 (3–10, 1–16)‡

*IQR, interquartile range; CQ, chloroquine; PQ, primaquine; Q, quinine; IV, intravenous; AL, artemether/lumefantrine; SP, sulfamethoxazole/pyrimethamine; AM, artesunate/mefloquine. †Values are mean (IQR, range) by category or days by patient. ‡p value = 0.009 for days until negative smear for *P. falciparum* vs. *P. knowlesi*.

Children with *P. falciparum* malaria were given a variety of treatment regimens. Most (71%) received a regimen that contained quinine, 7 (50%) children received sulfadoxine/pyrimethamine, 6 (43%) received primaquine, and 5 (36%) were given chloroquine. Only 5 (36%) received artemisinin-based combination therapy. Parasite clearance times were significantly slower among children with *P. falciparum* malaria, with a median of 5 days until the first negative smear; in 4 children (29%), it took >10 days for parasites be cleared. Children with *P. knowlesi* malaria had significantly shorter hospital admissions (median 4 days, IQR 4–5 days) than did children with *P. falciparum* malaria (median 7 days, IQR 5–10 days).

## Discussion

*P. knowlesi* was the most common cause of malaria in adults and children in the Kudat region in Sabah, Malaysian Borneo. Although those with *P. knowlesi* infections had an older age distribution than did those with *P. falciparum* infections, the species still caused 63% of all malaria cases among children <15 years. Although nearly all previous reported cases of knowlesi malaria have been in adults, 14% of all knowlesi cases in Kudat occurred in children. In children, *P. knowlesi* most often caused uncomplicated malaria, which responded well to chloroquine. Nevertheless, knowlesi malaria was associated with substantial illness in children, with all PCR-confirmed *P. knowlesi*–infected children being anemic at admission or during hospital stay.

In the only other report of knowlesi malaria in children, a prospective study of adult knowlesi malaria in Sarawak reported the exclusion of 8 children <15 years with *P. knowlesi* infection, comprising 7% of all knowlesi cases (*3*). The clinical and laboratory features of *P. knowlesi* malaria in children have not been described. Consistent with the reported features of *P. knowlesi* malaria in adults (*3*), the disease in most children was uncomplicated. In adults with knowlesi malaria, increasing age is a risk factor for severe disease. Although the numbers are small, none of the children had severe manifestations of knowlesi malaria that have been reported in adults (*3*), such as acute lung injury or acute renal failure. In falciparum malaria, these conditions are also largely confined to adults (*21*); anemia, coma, and acidosis-related respiratory distress are the major manifestations of severe falciparum malaria in children. No child with knowlesi malaria exhibited coma or respiratory distress; however, anemia developed in all children with knowlesi malaria, with the hemoglobin concentration in 1 patient (6%) falling to <7.0 g/dL. Anemia was more common in children than has been previously described in knowlesi malaria in adults (*3*).

Thrombocytopenia was found at admission in nearly all (94%) children with *P. knowlesi* malaria, in contrast to only half of the children with *P. falciparum* malaria. Although the cause is unclear, thrombocytopenia is also nearly universal in infected adults (*3*), which makes it a characteristic feature of *P. knowlesi* infection across all age groups. The role of thrombocytopenia and platelet activation in the pathogenesis of knowlesi malaria requires further investigation.

Most children with *P. knowlesi* malaria had an adequate response to a 3-day regimen of treatment with chloroquine and primaquine, although the mean parasite clearance time of 2 days was longer than the 90% parasite clearance time of 10.3 hours that was recently reported in adults (*25*). One child who received chloroquine and primaquine, and had a high parasite count at admission, required 5 days to clear parasites. Standard Ministry of Health pediatric dosing regimens of chloroquine are used in Kudat; however, posttreatment vomiting or inadequate blood concentrations could not be excluded. Prospective studies that evaluate the response to chloroquine and artemisinin-based combination therapy in pediatric knowlesi malaria are required. Children with *P. falciparum* malaria received many different treatment regimens and took significantly longer to clear their parasites. Less than half received the recommended artemisinin-based combination therapy, and only after alternative treatments failed. Children with *P. falciparum* malaria had significantly longer hospital stays, likely related at least in part to suboptimal treatment regimens. This finding highlights the importance of increasing the usage of artemisinin-based combination therapy for falciparum malaria in district hospitals in Sabah and elsewhere.

Our study had several limitations. First, PCR was only performed for 73% of cases across all age groups, and this limited our ability to accurately determine the true proportion of disease caused by *P. knowlesi*. Furthermore, although PCR was performed on samples from most children with *P. knowlesi* malaria, only 1 child with *P. falciparum* malaria had PCR performed. Our analysis, therefore, compared children with PCR-confirmed knowlesi malaria to children with falciparum malaria diagnosed by either microscopy or PCR. Some of those children with a diagnosis of falciparum malaria may have actually had knowlesi malaria, and the differences found between these 2 species may therefore be minimum estimates. The retrospective design of our study also limited our ability to collect standardized clinical information.

This study demonstrates that *P. knowlesi* has become the predominant malaria species in the Kudat region, estimated by results of microscopy, PCR, or both, as contributing to 87% of all malaria cases, a higher proportion than that reported elsewhere in Malaysian Borneo. The emerging dominance of *P. knowlesi* in Malaysian Borneo has been hypothesized to result from the following factors: changing patterns of human exposure to monkeys and vectors (*26*) because of deforestation, and potentially reduced competition or cross-species protection from *P. vivax* and *P. falciparum* as a result of a 40-fold reduction in the prevalence of these species in Sabah and Sarawak during 1960–2006 following intensive malaria control efforts (*27*). The paucity of *P. vivax* in Kudat was particularly notable.

Previous reports of adult disease have been from communities adjacent to rainforests (*3*). Kudat is a coastal rural farming area with varied land use and vegetation patterns and with minimal remaining regrowth forest. Although our retrospective study did not gather detailed travel or exposure histories, it is likely that most pediatric infections were locally acquired and that infections with *P. knowlesi* did not occur solely in those spending time in forested areas. Macaque monkeys, the natural hosts of *P. knowlesi*, are widely distributed in different habitats throughout the Kudat area and are frequently domesticated. The major vectors of *P. knowlesi* in forested areas of Sarawak, *Anopheles latens* mosquitoes, disappear with deforestation, but vectors capable of transmitting *P. knowlesi, An. balabacencis* mosquitoes (*26*), do persist at lower densities in largely deforested areas of Sabah (*28**,**29*). In notable contrast to *P. falciparum* malaria, pediatric knowlesi malaria was restricted to children of school age. Further studies will be required to characterize the transmission patterns, vectors, and risk factors for *P. knowlesi* in deforested areas of Malaysia.

## Conclusions

*P. knowlesi* is the most common cause of malaria in adults and children in the Kudat region of Sabah, a rural coastal deforested region. Consistent with previous studies in adults (*3*), we found that *P. knowlesi* in children most often caused uncomplicated malaria that responded adequately to chloroquine and primaquine. Anemia was common in children and knowlesi infection was associated with moderately severe anemia. Thrombocytopenia was universal and is characteristic of knowlesi malaria across all age groups. Larger prospective clinical studies are needed to describe more fully the epidemiology of *P. knowlesi* malaria in children, the full spectrum of clinical disease and the transmission patterns in nonforested areas such as Kudat.
